# How do community health actors explain their roles? Exploring the roles of community health actors in promoting maternal health services in rural Ethiopia

**DOI:** 10.1186/s12913-019-4546-7

**Published:** 2019-10-21

**Authors:** Abebe Mamo, Sudhakar Morankar, Shifera Asfaw, Nicole Bergen, Manisha A. Kulkarni, Lakew Abebe, Ronald Labonté, Zewdie Birhanu, Muluemebet Abera

**Affiliations:** 1 Department of Health, Behavior and Society, Faculty of Public Health, Institutes of Health, Jimma University, Jimma, Ethiopia; 2 Faculty of Health Sciences, University of Ottawa, Ottawa, Canada; 3 Schools of Epidemiology and Public Health, University of Ottawa, Ottawa, Canada; 4 Department of population and Family health, Faculty of Public Health, Institutes of Health, Jimma University, Jimma, Ethiopia

**Keywords:** Community health actors, ANC, Childbirth, PNC, Rural, Ethiopia

## Abstract

**Background:**

Maternal and child morbidity and mortality remains one of the most important public health challenges in developing countries. In rural settings, the promotion of household and community health practices through health extension workers in collaboration with other community members is among the key strategies to improve maternal and child health. Little has been studied on the actual roles and contributions of various individuals and groups to date, especially in the rural areas of Ethiopia. In this study, we explored the role played by different actors in promoting ANC, childbirth and early PNC services, and mainly designed to inform a community based Information, Education & Communication intervention in rural Ethiopia.

**Methods:**

An exploratory qualitative study was conducted on 24 in-depth interviews with health extension workers, religious leaders, women developmental army leaders, and selected community members; and 12 focus group discussions, six with female and six with male community members. Data was captured using voice recorders and field notes and transcribed verbatim in English, and analyzed using Atlas.ti software. Ethical approval for the fieldwork was obtained from Jimma University and the University of Ottawa.

**Results:**

Participants described different roles and responsibilities that individuals and groups have in promoting maternal/child health, as well as the perceived roles of family members/husband. Commonly identified roles included promotion of health care services; provision of continuous support during pregnancy, labour and postnatal care; and serving as a link between the community and the health system. Participants also felt unable to fully engage in their identified roles, describing several challenges existing within both the health system and the community.

**Conclusions:**

Involvement of different actors based on their areas of focus could contribute to community members receiving health information from people they trust more, which in turn is likely to increase use of services. Therefore, if our IEC interventions focus on overcoming challenges that limit actors’ abilities to engage effectively in promoting use of MCH services, it will be feasible and effective in rural settings, and these actors can become an epicenter in providing community based intervention in using ANC, childbirth and early PNC services.

## Background

**Fig. 1:**
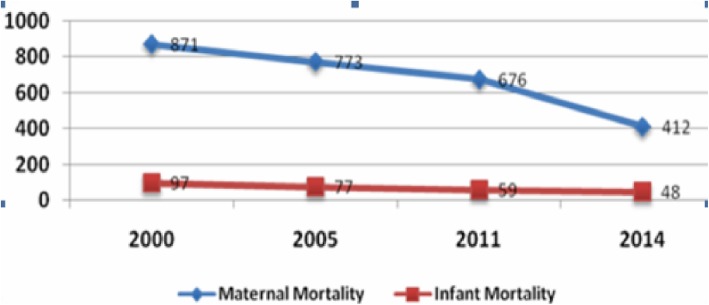
Fifteen years trends of maternal and health services in Ethiopia, 2016

Ethiopia is a low-income country with a total population of more than 90 million [1]. Its health status by most indicators ranks amongst the worst globally, even when compared to those for most Sub-Saharan Africa countries [2]. In the last two decades, the government of Ethiopia has strengthened the health system by applying pro-poor policies and strategies including the Health Extension Program (HEP) (2004), Child Survival Strategy (2015), Adolescent and Youth Reproductive Health Strategy (2005), and Ethiopia Hospital Reform Initiative (2010) [3]. These initiatives resulted in significant gains in the health status of citizens, with Ethiopia performing well in meeting most of the Millennium Development Goal (MDG) targets [2], including a steady decline in the maternal and infant mortality rates following introduction of the HEP [4] (Fig. 1).

The HEP depends primarily on Health Extension Workers (HEWs), a female cadre of salaried community health workers (CHWs) trained on 18 modules that comprise the HEP and its four components: disease prevention and control, family health, hygiene and environmental sanitation, and health education and communication [3]. The HEWs have been successful in reaching rural communities and improving MCH services [4]. HEWs work in close relationship with community members notably the Women Development Army leaders (WDA), a group of female volunteers who promote health within their local communities with an emphasis on health promotion, health education, and learning from others. WDA leaders are appointed from one of a cluster of five households and receive training from HEWs to function as ‘model families [3, 5]. The Male Developmental Army (MDA) was introduced in 2003, based on gradual training of male model household heads by agricultural extension agents to work on agricultural activities and a few public health areas largely associated with sanitation. Following successful implementation of the MDA, the WDA was introduced in 2012 with the aim of supporting HEWs in the implementation of the HEP, and replacing other community-based health workers such as health promoters and traditional birth attendants (TBAs). The major responsibility of the WDA was ensuring that the five households for which they are responsible are aware of and follow the health practices associated with the HEP program areas [2, 3]. Both the WDA and MDA are expected to take practical actions for health improvement at the individual, family, and community levels.

HEWs, together with WDA leaders, play a major role in extending health service coverage, contributing to an increased uptake of family planning, immunization, antenatal care, and HIV testing [4]. Outcomes in maternal health care, antenatal care (ANC), skilled delivery, and postnatal check-ups, however, remain poor [3], and Ethiopia’s MCH service utilization remains low with just over a quarter of pregnant women delivering with a skilled birth attendant at a health facility^1^in 2016. ANC coverage was also very low with only 32% of women receiving ANC four^2^and above, with only 62% receiving ANC services from skilled providers [6]. Similarly, a large proportion of maternal and neonatal deaths occur during the first 48 h after delivery, yet only 17% of women report receiving postnatal care (PNC) in the first 2 days after their last delivery [4]. Therefore, poor service utilization and home delivery is not only common, but also a custom for most women in Ethiopia, and according to EDHS 2016, 73% of live births in the 5 years before the survey were delivered in home [4]. It also mentioned that home delivery could be due to lack of awareness of the importance of skilled deliveries at a health facility, cultural beliefs, and the difficulties of transport in rural areas. Still many rural communities believe that home births are just as safe as health facility delivery plus feeling more comfortable at home. The problem is during home delivery there are no special supports or not safe delivery except that traditional birth attendants (TBAs) use their long experience in assisting delivery using different traditional methods like massaging the abdomen of pregnant women with butter, use unclean clothes and unsterilized razor, in which all of these supports and materials are very dangerous and mostly life threatening; so most of these factors have yet to be studied well in Ethiopia [3].

Although utilization of MCH services can be enhanced through available community-based interventions that are feasible to implement in resource-poor settings [4], community based intervention coverage in Ethiopia remains low [6]. Within existing coverage, Behavior Change Communication (BCC) activities have been key strategies used to improve utilization of MCH services in Ethiopia.

As part of larger research project, ‘An Implementation Study of Interventions to Promote Safe Motherhood in Jimma Zone, Ethiopia,’ one of the interventions will test an Information, Education, and Communication (IEC) strategy aimed at enhancing community- level behaviors supportive of the MCH program particularly on ANC, childbirth and early postnatal cares. At present, however, in-depth evidence is lacking on the actual roles, responsibilities, and contribution of different community individuals or groups in promoting such services. The project team thus identified HEWs, religious leaders, WDA leaders, MDA leaders, and other community members as the potential actors with a potentially important role in promoting ANC, childbirth and early postnatal cares. To support the preparation for the design and delivery of the IEC intervention, in this article we explored a qualitative study to ascertain the actual roles, responsibilities, and contribution of different community individuals or groups in promoting ANC, childbirth and early postnatal cares.

## Methods

### Study setting and period

The study was conducted in Jimma zone; an area located 356 km from Addis Ababa in Southwest Ethiopia, Oromia National Regional State. Jimma Zone has 21 districts, and 42 urban and 513 rural kebeles.^3^ The total population of Jimma Zone is estimated to be 3.2 million with the majority of the population living in rural areas [7]. This study area was selected based on the assessments by Jimma Zonal Health Department, MCH service utilization in the study districts indicated that with high population than other districts, the majority fall into the ‘least functional’ category and lack such basic facilities as water, bathrooms and beds [7]. There is a need to improve MCH service use and also need to be adequately integrated into the health system as part of the continuum of maternal and child health care.

### Study design

An exploratory qualitative case study was conducted in May 2016 in Jimma Zone to obtain information on the actual role, responsibilities and contributions of different actors in promoting ANC, childbirth and early PNC services, to assist in developing the proposed IEC intervention.

### Participant sampling and recruitment

Purposive sampling was used which involves selecting participants who share particular characteristics and have the potential to provide rich, relevant and diverse data pertinent to the research question, but we also included participants from remotest and closet villages from their respective PHCUs. The populations of interest were HEWs, religious leaders, Women Developmental Army leaders (WDA), Male Developmental Army leaders (MDA) and married male and female community members who have no any position in the community. We conducted 12 focus group discussions (FGDs) and 24 semi-structured, in-depth interviews (IDIs). FGD participants included female and male community members (Table 1).

**Table 1:** Overview of sampling for focused group discussions (FGD) and In-depth Interviews in rural Ethiopia, May 2016

Method	Participant type	No. per kebele	No. per district	Across three districts
FGDs	Female community members	1	2	6
	Male community members	1	2	6
	Total FGDs:	2	4	12
In-depth	WDA leaders	1	2	6
Interview				
	MDA leaders	1	2	6
	HEWs	1	2	6
	Religious leaders	1	2	6
	Total IDIs:	4	8	24
	Total FGDs and IDIs:			36

### Data collection

Nine bilingual individuals (seven males and two females) were employed to conduct data collection, including graduate students (MPH) and faculty members from Jimma University (Assistant Professors) who received 1 week training course in qualitative data collection. Semi-structured topic guides were developed in English and translated into Amharic and Afan Oromo languages. The topic guides were piloted in an area that was not included in the study. The FGDs and interviews included questions on demographic information, expected and actual tasks, responsibility, major contributions, or success, career, experiences relating to maternal health, challenges and suggestion (See Additional file 1). Further, we collected data on the perceived roles of husbands and other family members, as well as any other person identified as helping to address problems during pregnancy, childbirth, and after delivery. Two interviewers (one facilitator and a reporter) with extensive experiences in conducting FGD were assigned to conduct all FGDs in health posts and health centers. Two researchers from the Safe Motherhood Project were present in a supervisory capacity during data collection, and all FGDs and IDIs were audio recorded and transcribed into English by the assigned data collectors. A sample of transcript was randomly checked against the recordings by one bilingual researcher. Daily debriefing sessions with the researchers and all data collectors were held to discuss key findings, refine the IDI and FGD guides, identify saturation of themes, and refine lines of inquiry. To document their experiences and impressions, data collectors and researchers kept field notes. The duration of data collection for most FGD and IDI were 2 h and 1 h respectively. Before the interview all data collectors invested sufficient time in observing and discussion on general issues in the field and participants were encouraged to attain vignettes and support their detailed statements with examples, in order to understand both the overall context and their own experiences. Finally, researchers conducted an exit interview with each data collector to further probe the content of the field notes and debriefing sessions.

### Data analysis

For data analysis, the audio-recorded interviews and FGDs were transcribed and translated into English by the interviewers. Initial analysis of the interviews began during the fieldwork, when the interviewers wrote fieldwork reports for their supervisors. Coding was carried out through reading and re-reading the compiled transcripts using Atlas.ti software. Before actual coding began, the transcripts were independently read by Nicole Bergen (NB) and Abebe Mamo (AM) to identify key themes and develop a code book. Transcript coding was undertaken by three members of the research team: NB, AM and Shifera Asfaw (SA). To enhance inter-coder reliability, the coders each independently applied the code book to a selected and rich transcript and reviewed any differences in their coding, which were discussed and resolved. A final version of the code book was then developed leading to identification of themes, and sub-themes. Coded transcripts were further analyzed and summarized in narratives for each theme and sub-theme. Study findings were presented, discussed, and validated in a stakeholder meetings conducted in regional and district health offices.

## Results

### Description of socio-demographic characteristics

All HEWs were female and the average age of respondents was about 26 years. The average years of service was around 8 years, suggesting that many HEWs began working shortly after completing secondary school (a minimum requirement to become an HEW). As MDA were organized at 2003 9 years before the development of the WDA, the average age of MDA is much greater than WDA leaders. In this study the average age of MDA is 47 years and the mean years of service was about 13 years. The average age of WDA members in this sample is 38 years and the mean years of service is roughly five ranging from 2 to 6 years. All religious leaders were Muslims with minimum age of 36, maximum of 70 years, the average years of service was around 14 years, and all were male.

### Roles and contributions of participants

Participants were asked about their own roles, perceived roles of others, and the interaction among these participants, as well as their major contributions and challenges throughout their activities. The findings showed that different actors have a coherent understanding with the roles and responsibilities of the government policy, and they tended to focus on a few main roles that fall under the following four broad themes: promotion of health care services, provision of continuous support during pregnancy, labour and PNC, and working as a link between communities and the health system (Table 2).

**Table 2:** Description of major roles of community health actors in rural Ethiopia, 2016

Major Themes/roles	Sub-Themes/Categories	Specific activities undertaken under each role
Promotion of health care services	✓ Provision of Information, Education, and Communication (IEC) ✓ Provision of Health Care Services	The most commonly cited role identified by all participants was provision of information, provision of preventive and curative health care services. WDA leaders are good in passing different knowledge on to mothers and members of the community during community meetings, women’s association meetings, antenatal outreach sessions, and coffee ceremony. HEWs, WDA and religious leaders are also participating on community mobilization activities including use of full ANC services, health facility delivery and PNC including the promotion of breastfeeding and child nutrition, immunization, and related matters.
Provision of continuous support	✓ Assistance for Health Services ✓ Assistance with social supports ✓ Supporting the Community Referral System	Activities identified as involving such support included assistance with community fund raising, facilitating ambulance services or traditional ambulances to get women to the health center for delivery, providing training for model family/WDA, and offering social support (practical help with routine activities, resources and material goods, emotional support and assurance, nutritional support, and accompaniment).
Work as community - Health Care System Linkage	✓ Identification, Registration, and Notification of Women ✓ Training, Supervision and Report	Integrating activities between community leaders, including WDA leaders, religious leaders and HEWs, are all considered to be bridges and enhance strong relationship and communication between HEWs, primary health care units and community members. WDA leaders are well in assisting HEWs in community mobilization, health education, identification, registration, and notification of pregnant women and newborns. This support was also strengthened by training, supervision and monthly, quarterly or yearly reports.
Challenges in promoting safe motherhood in community	✓ Challenges from Health System Side ✓ Challenges from community Side	Most participants expressed concerns on the poor quality of MCH services like substandard quality of care, lack of teaching resources, and lack of incentives for undertaking home visits in remote areas were not motivating. Further when these barriers are combined by poor community and men participation and some religious myths, they work as promoters of home delivery and barriers in promoting utilization of MCH services.

#### Promotion of health care services

The most commonly cited role identified by all participants is that of health promoter. The activities under this role were provision of information, provision of preventive and curative health care services, and community mobilization activities with the aim of encouraging particular behaviours, including use of health care services during pregnancy, childbirth, and after childbirth.

##### Provision of information, education, and communication (IEC)

Providing IEC that promoted use of maternal health care services was regarded as an important role of all participants. Participants identified the health developmental army structure and husbands/neighbors as the main means for IEC, which included a range of topics related to antenatal care, birth preparedness, vaccinations, use of Maternal Waiting Area (MWA), importance of giving birth at the facility, and postnatal care (child immunization, exclusive breastfeeding, and regular check-ups). To implement these activities the HEWs and WDA leaders used different strategies such as home visits and family conversation, held weekly community meetings, and sometimes undertook community mobilizations. These approaches gave WDA and HEWs an opportunity to discuss with all family members and inspect homes on families’ progress in implementing the key HEP messages on ANC, MWA, childbirth and PNC.

*“As women reach the fifth month of pregnancy we will have a discussion with her (pregnant mother) and advise her to visit health facilities, get immunized, and visit the health facility ---, we advise women to use Maternal Waiting Area and get rest by waiting there until the delivery date---- they can get good rest.”* (WDA from Gomma district).

Religious leaders are regarded as influential in many aspects of community life, and identified several roles and responsibilities they had to promote safe motherhood.

*“---- Now we are advising pregnant women to give birth in health facility. We also advise them to attend health checkup during pregnancy and go directly to health facility when labour starts. We are advising people to stop harmful practice like massaging the belly of pregnant women*”. (Muslim religious leader from Seka District).

##### Provision of health care services

Another role participants identified involves organizing and provision of preventive cares, diagnosis and management of common problems in promoting health care services. The provision role is mainly carried out by HEWs, based on 18 health extension packages that form the HEP. Other community members are not expected to provide these services, but they cooperate and organize with HEWs to ensure that they are provided. HEWs specifically mentioned working to improve HIV counseling and testing “*to prevent mother to child transmission”* (HEW from Kersa District). Another role mainly undertaken by HEWs involves the provision of health care, specifically different aspects of check-up for further treatment or assistance to women during labour and birth. The HEWs’ major tasks here commonly include detection of high-risk pregnancies and labour complications, the management of uncomplicated pregnancy, PNC, and diagnosis and management of common childhood illnesses. As a male FGD participant from Kersa district described, *“Starting from her last menstrual period, the health extension worker was there, for check-up as well as to provide her vaccine and supplement iron”*.

Health Developmental Army leaders (MDA and WDA) also noted that HEWs are providing diagnosis and treatment services for common health problems that might arise during pregnancy, such as hypertension and anemia.

*“Before and during pregnancy... women come to HEWs. If they have problem of blood pressure, the HEWs will give them medicine for that, they provide vaccination, they may also refer them to other health care”*. (Female FGD participants from Kersa District).

Despite the HEWs were not supposed to provide curative services for severe problems, they can provide different essential newborn care services.

*“On the child health we give them immunization. Before this within first forty five days we observe them whether they suck breast properly, cleanness of cord, measure their weight by using infant weighting scale, and (observe) how they keep them warm and their hygiene during home visit”*. (HEW from Seka District).

#### Provision of continuous support

Women participants said that they expected to have some problems during labour and delivery; but also they expected to receive culturally appropriate support to help them control and manage their problems. This view was widely shared amongst all participants, including the provision of continuous support throughout pregnancy, delivery, and after delivery. Activities identified as involving such support included assistance with community fund raising, facilitating ambulance services or traditional ambulances to get women to the health center for delivery, providing training for model family/WDA, and offering social support (practical help with routine activities, resources and material goods, emotional support and assurance, nutritional support, and accompaniment).

##### Assistance for health services

Most informants were quick to point out social and cultural factors that reinforce the importance of their involvement in promoting MCH and encouraging broader community involvement. Muslim religious leaders, for example, explained that the ethic of communal support was integral to their faith and leveraged this ethic to mobilize the community in assisting pregnant women in accessing the health facility when in labour. This includes encouraging the community to raise funds, or even helping ill individuals to get to the health facility when they faced financial or other constraints.

“*What I should do for pregnant women during pregnancy is taking them to health facility for regular check-up and helping them to go and deliver in health facility and taking the children to health facility for vaccination—“*. (Muslim Religious leader from Kersa district)

Fund raising within the community to support pregnant women and health-facility deliveries is also described as something that community leaders and other members are involved with. In some kebeles, funds are used for income generation purposes such as buying cattle. WDA and MDA leaders mentioned the existence of other, long-standing community support mechanisms, not associated with the government program. These supports function together with the WDA, or in place of it.

*“The women health development army exists in the form of AFOSHA (cultural self-help system). They help one another not only when someone dies, but also when someone gives birth and during festivals. They even help one another financially. They contribute money and buy basic household materials.”* (Male FGD participant from Gomma district).

Although the mandate of the MDA focuses on sanitation and agricultural development, MDA.

participants also stated that they are involved in assisting women’s health by facilitating supports on birth preparedness, providing transportation to health facilities, and speaking to husbands to support their wives. As one MDA member spoke about his role in helping women to prepare for delivery:

*“We are doing many activities like advising women to prepare before delivery. There could be different problems during delivery and they may need money, so I inform them to save certain money and keep it with them. ....... If I hear that someone is in labour, we are all running to her home and call ambulance. If there is no ambulance service we use human power to carry the woman to the health facility. This is the responsibility of each community member* ***.”*** (MDA from Gomma district).

##### Provision of social support

Participants described being accountable in supporting their community including accompaniment outside the homestead, practical help with routine activities, emotional/spiritual support and material aids. Some MDA leaders explained that they often accompany women in labour to the health facility and after a woman has given birth, WDA leaders provide assistance by helping to prepare food and facilitate family support with household chores.

*“...when she is ready to deliver, I will take her to the health center and then come back home with her... after delivery I am responsible for preparing food and giving her advice about not working beyond her capacity and for washing the baby clothes.”* (WDA leader from Seka district).

Different FGD participants also described having responsibility for support that extended to easing pregnant women’s burdens in both indirect and direct ways.

*“In our community women are doing activities like collecting firewood, fetching water and etc. So during pregnancy we have to exempt her from these workloads and provide her good food and support her to visit health facility. After delivery, it is good to provide her additional food; if possible, it is good to provide meat to replace what she missed while she was pregnant. During delivery, I have to go with her and motivate her to have healthy delivery. After delivery it is enjoyment and all family members can enjoy and provide all important things for her and new borne”.* (Male FGD participant from Gomma District)

Community members also explained how religious leaders are very important in providing emotional and spiritual support to their followers. They pray for people during hard times, such as when women and children are ill. These leaders warn husbands to support their wives by citing religious texts. This is also reported to be true for Christian churches and religious leaders as well, as a female community member explained:

“*In the Orthodox Church while the religious leaders pray, they also pray for pregnant women and for women in postpartum period ---- to live in agreements as our governments ordered us the church also ordered us too and made pray for us, we also pray at home.”* (Female FGD participant from Gomma district)

Other community members expressed ideas on how males, particularly husbands, can or should facilitate and support the general well-being of pregnant women, by helping with strenuous chores, encouraging pregnant mothers to seek MCH services, ensuring that the women sleep under bed nets, and providing appropriate food.

*“For pregnant mother, important thing is supporting them, fed them balanced diet and care for them. They have to eat various vitamins rich foods, not to carry heavy load and support them by making fire wood. The given care should be continuous up to they gave birth and also during their postnatal period”.* (Male FGD participant from Gomma District))

##### Supporting the community referral system

HEWs are expected to facilitate a safe and clean environment for birth, provide essential newborn care, and to recognize and refer women who have complications during pregnancy, labour, or after delivery. Similarly, community leaders also mentioned that they encourage institutional delivery by supporting the community referral system and organizing ambulances when needed. According to participants, when a woman goes into labour, the process of transporting her to a health facility may begin with carrying her on a traditional stretcher from her home to the main road on foot, if she lives away from the main road. An ambulance may also be called to take the woman to the health facility free of charge, or a private vehicle (“bajaj”) may be hired.

“*We have two ambulances and if the road is not comfortable for ambulances we can use bajaj and human power to transport pregnant women to health center for delivery. If it is suitable for ambulance services, what we are doing is just calling on their mobile phones from our home then the ambulance can come and take the mother to health center. So it is as simple as this if a pregnant mother likes to deliver at health center*.” (MDA from Seka Chekoresa district)

#### Work as a community-health care system linkage

Fostering a robust community-health system partnership in which both groups work strategically and collaboratively toward a common end offers the potential to strengthen service integration and enhance HEWs’ performance. Community leaders, including WDA leaders, religious leaders and HEWs, are all considered to be bridges between the community and the health system. Collaborative community health tasks identified by participants include fostering partnerships, strengthening linkages with various health and community system actors, and providing opportunities for HEWs to interact and support one another in their specific health system tasks. These tasks encompass identification, registration and notification of women during pregnancy, labour and after delivery; early recognition and referral of women who have complications during pregnancy, labour and after delivery; and provision of community-level training.

##### Identification, registration and notification of women

HEWs and WDA leaders are used to identify and register all women in the community who are pregnant or in labour or who have given birth, and to provide continuous follow up. Furthermore, WDA leaders explain that they are to notify the health extension worker of any new pregnancy, labour, or birth that they learn about through their community networks. Religious leaders recognized that, although mortality rests in the hands of God, accessing healthcare and acting upon advice provided by healthcare workers was important and not in conflict with any religious beliefs.

“*The role of the mosque is broad; the first one is advising the community to believe what health professionals order us to do is essential you die if you are ordered to die and live if you are ordered to live by God but you should believe the advice given by health professionals is true”.* (Muslim religious leader from Seka district)

WDA leaders also contribute to community records by telling the HEWs how many pregnant women they have in their villages and reporting on the extent of maternal health care service utilization. MDA leaders described their counterparts in the WDA as ‘special’ agents in supporting HEWs and promoting pregnancy and childbirth services.

*“What makes women health development army leader support special is that, they involve starting by enrolling the pregnant women and reporting to health extension worker at the termination of first menstrual cycle”.* (H EWs from Kersa district)

##### Training, supervision and report

HEWs mentioned that supervision is an opportunity to assess and improve their achievements, contributions and service delivery, and that supportive supervision encourages them to work better. In partnership with other community leaders, HEWs identify and train families to become role models to help diffuse health messages and adopt desired practices and behavior. HEWs recognize the WDA as a part of the community that is ideally placed to reach families effectively. Therefore, ensuring that the WDA is well-informed about key health promotion messages and assisting them with tracking changes in the community is a critical element in the HEW role.

*“....we taught them (the WDA) and raised their awareness and then made follow up to monitor the change they have brought about... (this) has helped improve the health status of the community”.* (HEW from Gomma District)

HEWs explained that working with religious leaders is important, with certain religious leaders having a direct role in promoting maternal and child health through actions such as increasing family planning utilization. As a HEW from Kersa district explained:

*“The religious leaders received health education from us and teach the community to use family planning and they helped us in solving the problem like increased family size or number of births which affects health of pregnant women and the new born baby”.*

HEWs are responsible for providing guidance and training on what advice WDA, MDA and religious leaders should provide to the community. They also receive reports from developmental army leaders.

*“Before the HEWs were assigned to our kebele we didn’t have any knowledge about the benefit of latrines and other health issues but after we received many trainings and (lots of) information. We discussed a lot of things with the HEWs like when to help pregnant mothers and how to inform them to deliver at health facilities....” (*MDA FGD participant from Seka Chekorsa district)

#### Challenges in promoting safe motherhood in community

Despite their acknowledged efforts to promote MCH services through strong interactions among HEWs, WDA leaders, religious leaders and wider community members, all participants reported that they faced different challenges from different sides.

##### The challenges from health system side

Most participants expressed concerns that supports from the health sector and the quality of MCH services were not motivating and that these were the major barriers to encouraging services use by pregnant women. A perceived substandard quality of care, the lack of teaching resources to support educational activities, and a lack of incentives for undertaking home visits in remote areas were all mentioned as major impediments to promoting MCH services. Participants also raised broader issues related to under-resourced health facilities and services, and their limited capacity to provide all services that women and infants need. They found it difficult to convince women of the importance of delivering at a health facility when the quality of care is substandard.

*“To be honest, we counseled pregnant women and refer them to the health center for delivery but when they go there (health facility) they don’t receive all the services that they need.”* (HEW from Gomma District)

WDA members reported a lack of basic infrastructure such as electricity, transport, and hygiene facilities, and poor service from health workers who often held negative attitudes towards patients. HEWs, although attributing the presence of the maternity waiting areas to the successful increase in the number of facility-based deliveries, complained that mistreatment by staff of MWA users caused huge barriers to promoting delivery at health facilities.

*“...when women have to give birth before the blood of another mother has been cleaned (at the health facility).......(it) encourages home delivery” .* (WDA from Kersa District)

##### The challenges from the community side

Participants mentioned that poor attendance at community meetings, limited opportunities to engage men/husbands, and some religious beliefs were some major barriers to community support in promoting utilization of MCH services. Organizing regular meetings with the community sometimes poses a challenge when kebele residents are reluctant to attend, which our participants attributed to members not always feeling that the meetings are beneficial. This is especially so when community members believe that no action has been taken on the requests they put forward at previous such meetings. An important barrier that WDA members reported facing in performing their duties is limited opportunities to engage with men or husbands, and conflicts in balancing their personal lives with the responsibilities that come with their position.

*“....my main problem is the overlapping responsibilities in the home and the community. I am expected to educate my children, care for my family, and serve the community simultaneously”. (*WDA Gomma District)

WDA leaders sometimes feel a lack of support from their husbands, with many husbands reportedly resistant to their wives joining the pregnant women groups or providing the financial resources necessary for family stuffs. WDA leaders also suggested that, while seeing improvements in the community because of their efforts is motivating, they would appreciate formal recognition by the government as well as some remuneration.

*“It will be good if I get some incentives while I work on these activities ...rather than always doing it for free.” (*WDA member, Gomma District)

Religious beliefs sometimes pose a challenge to facility-based birthing promoted by the community. Some families believe that the ultimate outcome depends on God’s will and it therefore makes no difference where a woman delivers. Successful home deliveries in the past also contribute to a reluctance to adopt this “modern” approach of facility-based births by some families.

7“*Our role is to see everybody who needs help but we have no budget for this but we asks who get sick as we can, a pregnant women when she get birth she stop coming to mosque. At this time we order the community to ask her and the community visit her, during their visit they give money for her we, we guides to continue helping each other but they doesn’t accept according to we order them, out often if three person accept us its good.” (*Religious leader, Seka Chekosa District)

## Discussion

Reducing maternal and child mortality relies upon community-based interventions that enhance cooperation and collaboration among diverse actors, especially in resource-poor settings [8]. Promoting such cooperation, in turn, rests upon effective information, education, and communication strategies. Culturally tailoring interventions have been shown to increase the likelihood that they will be favorably received and subsequently more effective at changing health behavior [9]. In keeping with this logic, and for our IEC intervention to be effective, we believed it important to precede it with a reasonably comprehensive evaluation of the customs and beliefs of the intended recipients/participants in the IEC, in order to create culturally and socially relevant health communication materials. As well, we believed it important to have a better understanding of the perceived roles, responsibilities, contributions, and major challenges of these community actors. In this respect our study is unique in incorporating various community members in identifying actual actors and contributions in promoting or providing MCH services.

Our study found that the most common role taken on by all participants is that of health promotion, and suggests that participants are quite uniform in the promotion of preventive health care services. Our results are in keeping with international findings [10, 11] and on the focus of the Ethiopian HEP program [12, 13] that underscore the importance of continuous provision of appropriate information using sound communication strategies and involving communities and households. Our study demonstrated the importance of HEWs and WDA leaders engaging directly with the community in general and pregnant women and their husbands in particular through home visits and counseling. For promotion of maternal and newborn health programs, WHO primarily recommends the use of CHWs, with a growing body of evidence concluding the promotion of health care behaviours and service utilization via CHWs, community members, and community opinion leaders is both feasible and effective [9, 14]. We can infer from this, and from our participants’ reported experiences, that strengthening the involvement of different community level actors (notably HEWs, WDA leaders, and religious leaders) based on their areas of focus and effectiveness will be influential in implementing context-specific maternal and child health interventions. Specifically, we anticipate that a skillfully developed IEC intervention should contribute to an increased use of ANC services, health facility delivery, and familial practices that promote MCH and postnatal care. An important aspect of our study is that it is grounded in the specific context of rural Ethiopia. Participants identified family members, community leaders, religious leaders, and HEWs as important sources of support. In line with this finding, other studies in developing-country settings where economic resources are scarce suggest that such local social support is both available and reliable, especially when the source of that support is family members or credible community leaders [15, 16]; when a substantial proportion of men provide a notable level of support to their wives during pregnancy [17]; and when faith leaders encourage the use of delivery and PNC services [18]. Patriarchal norms still predominate in Ethiopia, however, and especially in rural areas; as some of our participants noted. These norms position husbands as decision-makers who may choose not to support their wives in either promoting, or availing use of, MCH services. Pluralizing women’s support beyond just her own husband can help to overcome this barrier, and provide a base for stronger public health interventions that promote service utilization and the well-being of mothers, newborns and the whole family.

Our study also explained that HEWs, WDAs leaders, religious leaders, family members, and other community members are working closely to create better and more sustained linkages with the health system. The use of HEWs as agents of health promotion and using WDA leaders as their close allies embodies a well-known approach in community health and development programs [19, 20]. WDA leaders appear to be involved in most aspects the MCH program, and are generally seen as being very supportive of HEWs, especially in hard-to-reach areas. This finding is consistent with previous studies in Ethiopia which find that community support to HEWs is frequently provided by the voluntary WDA, churches, mosques, and community associations. This strong interaction amongst local community actors, which our study also affirmed, represents a strength that can be utilized in an IEC intervention to promote MCH service utilization.

As important as the roles and shared responsibilities are between communities as MCH promoters, there remain important challenges. Several of these emerged from the interviews reflecting challenges arising from both the health system and the community. Health system challenges identified by our participants focused mostly on the issue of quality: poor facilities, unhelpful behaviours by some of the health staff, unhygienic MWAs, and a lack of health facility resources, all of which lead to a limited capacity to provide the level of service that can motivate pregnant women to give birth in a health facility, or to use ANC and PNC services. Other studies similarly caution that poor supports and service resources can hamper health workers’ performance [15], again undermining trust in their relationships with the community [16]. HEW frustration extended to the inability of health facility staff to provide transport services for remote areas during home visit sessions and a lack of health learning materials, making it harder for them, and for the WDA, to educate women on the importance of delivering at a health facility. Although not a major issue identified by our participants, other Ethiopian studies find that health workers’ lack of competence in childbirth and PNC can negatively affect community trust in the health system [21]; quality concerns in the health system are likely to be issues affecting the MCH program for some time to come.

From the community side, poor attendance at community meetings, previous successful home delivery experiences, religious beliefs, and limited opportunities to engage men or husbands were thought to be the main challenges that needed to be addressed. The explanation for poor community participation in meetings (that their previous participation did not lead to any change or improvements) is consistent with other studies of community health workers’ efforts to mobilize communities [22–25] and emphasizes the importance of not only identifying health problems but also implementing new actions based on how communities identify their needs. Religious beliefs that the life or death of a mother and baby “depends on God’s will” and not birth preparedness or place of delivery is a common finding in other studies [14, 26, 27]. Some religious leaders, for example, are hesitant to cooperate on issues like family planning (believing it is a sin) or are opposed to vaccinations because they believe it interferes with divine providence. But studies also document that other religious leaders accept vaccination as a gift of God [24, 28] and are less opposed to family planning and identified aspects of their respective roles that encouraged a community approach [29]. Our small-scale study indicates that similar differences might exist amongst religious leaders in our intervention areas, but some of them also had useful suggestions for change, such as locating MCH supportive passages in religious texts. Our own results, alongside those from other studies, suggest the importance of HEWs and WDAs working with those religious leaders who are comfortable with, for example, family planning and vaccination, to convince others religious leaders of the importance of such services.

### Limitation of the study

This study has the following limitations. First, this study was unable to explore information from zonal health officers to understand the clear roles and the relationships between health professionals and HEW supervisors, HEWs and their communities. However, we tried to include respondents from different settings and using triangulation of different types of respondents and data collection processes, the findings present useful information for understanding better the actual roles and contribution of community health actors in promoting MCH services.

Second, athough we tried to include different participants from different sites as well as encapsulated major emerged themes in promoting the use of ANC, childbirth and early postnatal care services, some components of the MCH services like prevention, identification and treatment of child illness, as well as specific distance from a woman’s residence to a health facility were not explicitly accounted in this study.

Third, due to the fact that this study tried to explore the actual roles of the participants in promoting the use of ANC, childbirth and early postnatal care services, obtaining an honest response from most HEWs and developmental army leaders could be difficult and there may be social desirability bias so that they may hide the real performance. To ensure such bias the authors explained about the objectives of the research and ethical issues related to safety, confidentiality, and privacy issues to all participants in the local language before started the interview or discussion.

## Conclusion

This study provides the primary health care programme with a better understanding of how actors within that program perceive their roles and shared contributions. It points to certain strengths that can be built upon, while also identifying ongoing challenges that the program, and notably our planned IEC intervention, need to address. HEWs and WDA leaders are clearly seen as the epicenter of community health, particularly for promotion of MCH services, and in functioning as a bridge between communities and the health sector. Most participants thought that improving the involvement of husbands in promoting or supporting MCH services represents a big opportunity to increase the effectiveness of the HEP. Ongoing concerns about substandard quality of care, under-resourced health facilities, and poor service from health workers will need to be addressed. Any increase in service demand, which is the goal of the HEP, and of the IEC intervention that forms part of our larger research project for which this study is one small component, should be matched by an increase in service supply, to avoid communities losing trust in the program. At the same time, our participants clearly spoke to their own potential to make a major contribution in extending uptake of family planning, antenatal care, MWA utilization, and increased health facility delivery and use of PNC services; all of which have the potential to reduce significantly maternal and child morbidity and mortality. This finding suggests that community-based MCH strategies are feasible in rural Ethiopia and are likely to be effective.

## Supplementary information

**Additional file 1.** In-depth interview and focused group discussion guidelines for Implementation study of Interventions to promote safe motherhood by Jimma and Ottawa Universities collaboration Project in rural Ethiopia.

## Data Availability

All datasets used and/or analyzed during the current study are available on request from the Corresponding Author, and focused group discussions and In-depth interview guidelines were submitted with this manuscript as additional file.

## Abbreviations

ANC: Antenatal care
BCC: Behavior Change Communication
CHWs: Community Health Workers
EDHS: Ethiopian demographic and health survey
FGD: Focused Group Discussion
FMoH: Federal Ministry of Health
HDA: Health Development Army
HEP: Health Extension Program
HEWs: Health Extension Workers
HIV: Human Immune Virus
IDI: In-depth Interview
IDRC: International Development Research Centre
IEC: Information, Education and Communication
IMCHA: Interventions to Promote Safe Motherhood
MCH: Maternal and child health
MDA: Male Developmental Army
MDG: Millennium Development Goal
MMR: Maternal Mortality Rate
MWA: Maternity Waiting Area
PNC: Postnatal care
RDT: Rapid Diagnostic Testing
TB: tuberculosis
TBAs: Traditional Birth Attendants
WDA: Female Health Developmental Army
WDA: Women Development Army

## Footnotes

1 By health facility we mean either a health center or a hospital staffed with physicians, nurses, and/or midwives; but not health posts that are staffed only by HEWs.

2 ANC services consist of at least four separate visits. A present weakness in MCH services in Ethiopia is the low number of pregnant women who do not receive, or do not seek out, adequate ANC coverage

3 Kebele – the lowest government structure that with communities of about 5000 people served by one health post.
