# Supplementary material for: How do community health actors explain their roles? Exploring the roles of community health actors in promoting maternal health services in rural Ethiopia
Source: BMC Health Serv Res. 2019 Oct 21;19:724. doi: 10.1186/s12913-019-4546-7 (PMC6805355; doi:10.1186/s12913-019-4546-7)
Supplement: Supplementary file 1 — Additional file 1. In-depth interview and focused group discussion guidelines for Implementation study of Interventions to promote safe motherhood by Jimma and Ottawa Universities collaboration Project in rural Ethiopia. [file 12913_2019_4546_MOESM1_ESM.docx]

**Annex I: Additional files [FGD and IDI Guides]**

**Jimma University**

**Implementation study of Interventions to promote safe motherhood by JU-Ottawa University collaboration Project**

**In-Depth Interview guide for Health Extension Workers**

**Key Informants**

- **Health Extension Workers**

Back Ground information of Interviewee

- Name of District_____________________
- Name of Health center and Health post______________
- Age of interviewee__________________
- Responsibility of the HEW __________________
- **Time started __________________**

**In-depth interview Guide - HEWs**

1. How do you break down your time for maternal health services and other health extension programs? How much of your work week is for maternal and child health program?
2. Give a detailed list of maternal and child health services you provide for the community?
3. What do you do to encourage members of your community so that they participate in maternal and child related activities, education or planning?
4. What are some serious health problems that can occur **during pregnancy** that could endanger the life of a pregnant woman? Which of these problems are severe? Could a woman die from [this problem] any of these problems?
5. What are some serious health problems that can occur during **labour and childbirth** that could endanger the life of a pregnant woman? Which of these problems are severe? Could a woman die from [this problem] any of these problems?
6. What are some serious health problems that can occur during **postpartum period** that could endanger the life of a pregnant woman? Which of these problems are severe? Could a woman die from [this problem] any of these problems?
7. How many ANC attendants could you face each month on average?
8. What are the services given to pregnant women during antenatal care visits?
9. What was the level of ANC 1 to ANC 4 dropout rate? What are the reasons for high dropout rate?
10. How do you understand the function, quality of services and impact of maternal waiting area in reducing maternal and neonatal mortality?
11. What are the factors that discourage pregnant mothers not to stay at maternal waiting area before immediately close to their delivery? What are the solutions you suggest?
12. What are the roles of health extension workers to increase the uptake of Maternal Waiting Area?
13. What are the leading reasons why women not prefer to follow antenatal care services? What are the possible solution do you suggest to curb such problem?
14. In your community how do women prepare for birth? What birth preparedness related services are found in your community?
15. In your locality where do women prefer to give birth and to be assisted by? Why?
16. Top reasons why women prefer to give birth in a home rather than elsewhere?
17. Why all women do not seek delivery care at health facilities?
18. Do you think the health problems can arise after birth? What are the health problems that can happen during that period? do you think it is necessary? Do women get check up after birth for their health? Why**?**
19. Do pregnant women in your community seek care after their delivery? Where did they prefer to go? Why?
20. Could you name some types of basic care that can be provided to a newborn baby immediately after birth?
21. What are problems in your community that hinder pregnant women from getting health services childbirth/labor, and post partum period?
22. How do you involve men during household visit or health education concerning maternal and child health?
23. How do you understand preparation for birth? What practices are common in your community? Who is responsible for saving money? How do women get financial support?
24. How do you work together with health development army, opinion leaders and model households to promote the health of mother and to reduce maternal and neonatal mortality?
25. When did you refer pregnant women for ANC, Delivery services and PNC to upper level of health level of health system? Where? How?
26. What did you do after they return to the community?
27. How often health center offices visited your community pertaining to maternal and child health services? What they do? What does the guideline say about the visit
28. How do you get feedback from health center officers or district health offices regarding maternal and child health services? Does the feedback have values?
29. Do you have routine meeting with health development armies? In what ways community residents participate in maternal and child health activities?

- **Time ended __________________**

***Thank you for your time and great participation***

**Back Ground Information of Interviewer**

- 1. Name of Interviewer ___________________________
  2. Sex_________________________________________
  3. Age of Interviewer_____________________________
  4. Educational level _______________________________
  5. Date of Interview ___________________________; Signature _________________

**Jimma University**

**Implementation study of Interventions to promote safe motherhood by JU-Ottawa University collaboration Project**

**In-depth interview Guide (Questions) for Religious Leaders**

**Key Informants**

- **Religious Leaders**

Back Ground information of Interviewee

- Name of District_____________________
- Name of Health center______________
- Age of interviewee__________________
- Responsibility of the religious leader __________________
- **Time Started:____________________**

**In-depth interview Guide - RL**

1. What are the roles of religious leaders in promoting maternal and child health to decrease maternal and neonatal mortality and morbidity?
2. Dou you have a routine program with health development agent, male development agent and community members to discuss on the issue of maternal health? What mal –practices were changed through these discussions?
3. What are some serious health problems that can occur **during pregnancy** that could endanger the life of a pregnant woman? Which of these problems are severe? Could a woman die from [this problem] any of these problems?
4. What are some serious health problems that can occur during **labor and childbirth** that could endanger the life of a pregnant woman? Which of these problems are severe? Could a woman die from [this problem] any of these problems?
5. What are some serious health problems that can occur during **postpartum period** that could endanger the life of a pregnant woman? Which of these problems are severe? Could a woman die from [this problem] any of these problems?
6. What do women in your community do during their pregnancy? Any visit to health center/health post? How many times in total pregnant women should receive antenatal care during pregnancy?
7. What are the services given to pregnant women during antenatal care?
8. How do you understand the function, quality of services and impact of maternal waiting area in reducing maternal and neonatal mortality?
9. What are the factors that discourage pregnant mothers not to stay at maternal waiting area before immediately close to their delivery? What are the solutions you suggest?

1. What are the leading reasons why women not prefer to follow antenatal care services? What are the possible solution do you suggest to curb such problem?
2. In your community how do women prepare for birth? What birth preparedness related services are found in your community?
3. In your locality where do women prefer to give birth and to be assisted by? Why?
4. Top reasons why women prefer to give birth in a home rather than elsewhere?
5. In your community who will make final decision where the women give birth and birth assistance? why?
6. What is the nature & extent of husband`s involvement in decisions on the use of maternal and child health services? What happens if husbands disapprove seeking the care?
7. Why all women do not seek delivery care at health facilities?
8. Do you think the health problems can arise 2 days after birth? What are the health problems that can happen during that period? do you think it is necessary? do women get check up after birth for their health? why**?**
9. Do pregnant women in your community seek care after their delivery? Where did they prefer to go? Why?
10. Could you name some types of basic care that can be provided to a newborn baby immediately after birth?
11. In your community do women freely discuss pregnancy and childbirth matters? with whom? Why?
12. What are problems in your community that hinder pregnant women from getting health services childbirth/labor, and post partum period?
13. What preparations are found in your community for emergencies? do people make advance preparations? What barriers those hinder from such preparations?
14. What are the roles of health development army in promoting the health of mothers and in reducing maternal and neonatal death?
15. How do you do with health extension workers to promote the health of mother and to reduce maternal and neonatal mortality?

***Time Ended:____________________***

***Thank you for your time and great participation***

**Back Ground Information of Interviewer**

- 1. Name of Interviewer ___________________________
  2. Sex_________________________________________
  3. Age of Interviewer_____________________________
  4. Educational level _______________________________
  5. Date of Interview ___________________________; Signature _________________

**Jimma University**

**Implementation study of Interventions to promote safe motherhood by JU-Ottawa University collaboration Project**

**Focus group discussion Interview guide for Women’s Health Development Army (WDA)**

**Time started: ___________________________**

**Focus group discussion Interview guide - WDA**

1. Do you have regular meetings on the issue of ANC, delivery and PNC? How often do you meet each other? What are the roles of health development army in promoting the health of mothers and in reducing maternal and neonatal death?
2. How do you work together with health extension workers to promote the health of mother and to reduce maternal and neonatal mortality?
3. How do you access information on maternal and child health services in the community?
4. In your community do women freely discuss pregnancy and childbirth matters? With whom? Why? If not why not?
5. What do women in your community do during their pregnancy? Any visit to health center/health post?
6. What are some serious health problems that can occur **during pregnancy**? (Which of these problems are severe? Have you ever observed or witnessed in that any pregnant woman die from those serious health problems related to pregnancy? Have you tried anything to safe life of the woman/women? What and how or why not? Can you tell more?)
7. What are some serious health problems that can occur during **labour or childbirth? (Which of those health problems are severe? Have you observed or witnessed that any woman die of those problems during childbirth?** Anything tried to safe the life of the labouring mother/mothers that you observed? By whom? Where? How? And if not why not? Can you elaborate more on this issue what should be done by the pregnant woman? Husband and family? By the community? By you? And health institutions?)
8. What are some serious health problems that can occur during after delivery within one to six weeks that could endanger the woman/women? Which of these problems are severe? Have you encountered any of these problems? What were the immediate solution/s/ done in order of actions? If no action why?)
9. Do pregnant women in your community visit to health post or health centre during pregnancy for checkups? How many times they visit if they visit? For what purpose they visit if they visit? If they did not visit why not?
10. For those mothers who visited health post/health center for Antenatal care, what services are given to them? When or at what visit for which service/s/?
11. How do you judge the function, quality of services given during ANC? Why?
12. What about the quality and coverage of maternal waiting area service provision, (functionality and quality) to impact or reduce maternal and newborn death rates? (How? Why? Or why not? What else needs to be improved?)
13. What are the factors that promotes or discourage pregnant mothers to stay at maternal waiting area? What are the solutions you suggest?
14. What are the roles of health development armies in promoting the utilization of ANC and maternal waiting area? (Were there problems? How those problems were addressed? What else be in place? By who could be provided?)
15. Do pregnant women prefer to have ANC and get delivery at Maternal waiting areas currently? Why or why not? How can the issues be addressed? Who is responsible for what?)
16. In your community how do women prepare for birth? What birth preparedness related services are found at family and community?
17. Every action needs decision. So, who decided the place of delivery (either at home or health institution or attended by relatives or medical person?). Why? Were the decisions accepted at what circumstances?).
18. How likely the condition of service provision influence Decision Making? How and why? What do you recommend to have safe delivery (or delivery attended by midwife at health center)?
19. After the birth what happened to the child and mother? What did they do? Where did they go? Any visit to health center? Why/why not? What did they do? Any visit from HEW or Community volunteer? What did they do?
20. Do you think the health problems can arise 2 days after birth? What about within 7 days, 15 days 20 days, 30 days and so on?). When it be expected no health problem associated to childbirth for the mother and newborn?
21. When do mothers start breast feeding? How long did mother’s breast feed their baby? When do mothers start additional feeding to their baby? Why and why not?
22. When does the newborn get immunized or vaccinated? By whom? If not immunized, why? (Can you mention the benefits of vaccination to the newborn? What are those benefits? Are those benefits crucial to the survival of the newborn and the life afterwards? How and why? What is/are the values held by the mothers, community members in general about the benefits of getting vaccinated to the newborn? Why and why not? Discuss more on this topic
23. Did HEWs visit your home in the last one year: How frequently they visit your home? Where you think HEWs live? What services did they give you?
24. Who supervise your efforts and actions to safe the mothers and newborn babies in your community? Are you satisfied by your actions so far? Why or why not?
25. Anything you will add or recommend before we rewind our discussions-you are well come?

**Time Ended:____________________**

***Thank you for your time and great participation***

**Back Ground Information of Interviewer**

- 1. Name of Interviewer ___________________________
  2. Sex_________________________________________
  3. Age of Interviewer_____________________________
  4. Educational level _______________________________
  5. Date of Interview ___________________________; Signature _________________

**Jimma University**

**Implementation study of Interventions to promote safe motherhood by JU-Ottawa University collaboration Project**

**Focus group discussion Interview guide for Male Development Armies (MDAs)**

**Time started: __________________________________**

**Focus group discussion Interview guide - MDA**

1. How villagers talk about pregnancy and delivery?  (Planning to have baby, safety of the pregnancy, safety of the pregnant women? How and what? If not why not?
2. Do you have regular meetings to discuss on maternal and child health? How often do you meet? What are the roles of male development army in promoting the health of mothers and in reducing maternal and newborn death?
3. Are the male development armies working together with the Health Extension Workers to promote pregnant mothers and child health? What about working together with the Women Health Development Army? What and how? If not why not?
4. Where, when and how do you access information on pregnant woman and child health services in the community?
5. In your community do women freely discuss pregnancy and childbirth matters? With whom? Why? If not why not? What about husband whose wife is getting pregnant? To whom why? If not why not?
6. What does a husband do during the time when his wife becomes pregnant? Any visit to health center/health post together? Arranging transportation means? What else?
7. Could pregnant women in your community visit to health post or health centre during pregnancy for checkups? In average how many times do you think pregnant woman need to visit while pregnant?
8. At what month of the pregnancy period do pregnant women consult the health personnel for health services in your village? For what purpose they visit? If they do not visit why not?
9. For those mothers who visited health post/health center for health checkups, what services are given to them? When or at what visit for which service/s/?
10. How do you judge, (good, bad, or else), the function, quality of services given during the pregnant women visit to health institutions? Why?
11. What about the quality and coverage of maternal waiting area service provision, (functionality and quality) towards reducing pregnant women and newborn death rates? (How? Why? Or why not? What else needs to be improved?)
12. What are the factors that promotes or discourage pregnant mothers to stay at maternal waiting area immediately close to their delivery? What are the solutions you suggest?
13. What are the roles of development army in your village in promoting the utilization of maternal and child health services utilization as well as staying inside maternal waiting areas at least for a week or two weeks? (Are there problems? How those problems were addressed? What else is need in place? By who could be provided? Why? And how?)
14. Do husband prefers his pregnant wife to stay at maternal waiting areas for a week and so and get deliver? Why or why not? Do the pregnant women prefer to stay at Maternal Waiting Areas? Why and Why not? How can be improved or addressed? Who is responsible for what?)
15. In your community how do women prepare for birth? What birth preparedness related services are found at family and community?
16. Every action needs decision. So, who decided the place of delivery (either at home or health institution or attended by relatives or medical person?). Why? Were the decisions accepted at what circumstances?).
17. How likely the condition of service provision influence Decision Making? How and why? What do you recommend to have safe delivery (or delivery attended by midwife at health center)?
18. After delivery what are the immediate care for the mother and the newborn? What did they do? Where did they go? Any visit to health center? Why/why not? What did they do? Any visit from HEW or Community volunteer? What did they do?
19. Do you think the health problems can arise 2 days after birth? What about within 7 days, 15 days 20 days, 30 days and so on?). When do we be sure no health problem that affects the newborn and the mother who gave birth due to delivery?
20. What are the major serious health problems (during pregnancy, child labor, after delivery)?
21. Was there any pregnant woman die in your village from serious health problems during pregnancy, child labor or post labor period? If yes, what was/were tried to safe life of the woman? What and how? Can you elaborate more by whom, how and when? What else do you recommend to safe lives of pregnant women on such occasions? If not tried, why not?
22. When do mothers start breast feeding? How long did mother’s breast feed their baby? When do mothers start additional feeding to their baby? Why and why not? Do husband support child breast feeding and feeding? Why or why not?
23. Do the newborn get immunized or vaccinated? When and by whom? If not why? (Can you mention the benefits of vaccination to the newborn? What are those benefits? Are those benefits crucial to the survival of the newborn and the life afterwards? How and why? What is/are the values held by the mothers, husbands, community members in general about the benefits of getting vaccinated to the newborn? Why and why not? Discuss more on this topic
24. HEWs visit your home in the last one year: How frequently they visit your home? Where you think HEWs live? What services did they give you?
25. Who supervise your efforts and actions to safe the mothers and newborn babies in your community? Are you satisfied by your actions so far? Why or why not?
26. Anything you will add or recommend before we rewind our discussions-you are well come?

- **Time ended __________________**

***Thank you for your time and great participation***

**Back Ground Information of Interviewer**

- 1. Name of Interviewer ___________________________
  2. Sex_________________________________________
  3. Age of Interviewer_____________________________
  4. Educational level _______________________________
  5. Date of Interview ___________________________; Signature _________________

**Jimma University**

**Implementation study of Interventions to promote safe motherhood by JU-Ottawa University collaboration Project**

**Focus group discussion Interview guide for Married Women from Community**

Time Started: _____________________________

**Focus group discussion Interview guide – Married Women**

1. Let us discuss about mother and child, how you feel and think they should be cared? (Probe: By whom? Why? How?
2. Do you think mothers’ and children’s health care is important? (Probe: Why? Are they cared as we think and want? Why and how?
3. Do you tell me about ANC and its important?
4. Have pregnant mothers gone for ANC in your village or Kebele? (Probe: If so, where? How? Where do they prefer to go for ANC? Why?
5. Let’s discuss about some health problems the pregnant mothers may encounter during pregnancy, delivery and after delivery within weeks?
6. Have you ever seen or encountered any of those pregnancy and delivery related health problems? (Probe: Tell me more about why those problems happen? What was/were done? By whom? When (immediately or late)? Why? Whom do you think is highly responsible for prevention or averting these pregnancy and delivery related problems? About Maternal Waiting Areas and its advantage and disadvantages)
7. Now let’s discuss about the newborn care? How is the newborn cared while a mother gives birth? (What is going to be done for the new born? When? Where? By whom? Why? What about immunization, when to which vaccination?)
8. Do you feel and think religious leaders can play role in caring the mothers and children? (What, how, why? What should be done?)
9. What are the roles of husbands in caring pregnant mothers and newborn baby in your village? How? Why? Why not?
10. Was the new born get adequate care? If yes, what are the cares provided to the new born? When? By whom? If not, why? What else do you feel not provided?
11. Were the newborn vaccinations provided on time? When? Where? By Whom? Or why not?
12. Tell us what you perceive the new born to the mother? To the father? To the family? To the community and nation?
13. What do you feel or suggest important care to the newborn and how?
14. What do you think or view the responsibility of women, men, community, and government to give maximum health care for pregnant mothers and newborns? How? Why and why not? Put the recommendations specific.
15. Now we are on the way to windup but before we do so, anything to be added or left to be discussed from your side or you need to pass the message or any errand to anybody.

**Time Ended: ____________________**

***Thank you for your time and great participation***

**Back Ground Information of Interviewer**

- 1. Name of Interviewer ___________________________
  2. Sex_________________________________________
  3. Age of Interviewer_____________________________
  4. Educational level _______________________________
  5. Date of Interview ___________________________; Signature _________________

**Jimma University**

**Implementation study of Interventions to promote safe motherhood by JU-Ottawa University collaboration Project**

**Focus group discussion Interview guide for Married Men’s**

**Time started: _____________________**

**Focus group discussion Interview guide – Married Men**

1. How husbands and other family members view pregnancies? What do they do for pregnant women?
2. How do you describe the husband’s support to his pregnant wife (such as for having care of the foetus, to get ANC, Vaccination and other recommended medical care during pregnancy, to deliver at health institutions and so on)
3. What are the major roles of the husband when his wife get pregnant as you feel and think appropriate?
4. Do you notice or observed any of those supports be adequately provided to the pregnant women by her husband? (What? How? Why? Or Why not?)
5. What do you know about the pregnant woman does care her pregnancy? (where her practice appropriate? If not why?)
6. What is expected from other relatives other than husband when a woman becomes pregnant? Why?
7. Is husbands collectively support the Maternal Waiting stay for one to two weeks before her delivery? Why? Why not?
8. What are the contributions of the community to support Maternal Waiting Areas so far? (do you think this support is appropriate? If not appropriate what else do you think is appropriate? )
9. What are some serious health problems that can occur **during pregnancy**? Which of these problems are severe?
10. Have you observed while a woman die from pregnancy related health problems? From [this problem] any of these problems? (what was/were the problems?, was/were any attempt to save the pregnant woman/women’s life either by the community, traditional practitioner, bringing to the health institution, etc? So, why the attempt fail to safe the pregnant woman/women’s life.
11. In your community how do women prepare for birth? What birth preparedness related services are found in your community? (Probe: is there any traditional practice that the community consider important for pregnant woman and during delivery? Why it is important? Who brought the knowledge and practice first?
12. How do you view or judge the services provided during ANC to pregnant woman by the health institution? Is it adequate? What about the quality?
13. How you judge the health personnel service provision to pregnant woman? Do they service with dignity and autonomy? Do they support and educate well?
14. Was the new born get care adequately? If yes, what are the cares provided to the new born? When? By whom? If not, why? What else do you feel not provided?
15. Were the newborn vaccinations provided on time? When? Where? By Whom? Or why not?
16. Tell us what you perceive the new born to the mother? To the father? To the family? To the community and nation?
17. What do you feel or suggest important care to the newborn and how?
18. Now we are on the way to windup; but, if anything left to say or forward a message that is in your mind before we windup, you well come!

**Time Ended:____________________**

***Thank you for your time and great participation***

**Back Ground Information of Interviewer**

- 1. Name of Interviewer ___________________________
  2. Sex_________________________________________
  3. Age of Interviewer_____________________________
  4. Educational level _______________________________
  5. Date of Interview ___________________________; Signature _________________

**Participants Background information**

- **Setting/Kebele:**
- **Participant:**
- **Interviewer:**
- **Transcriber:**
- **Interview Code:**
- **Date:**
- **Duration:**
- **Code (I): Moderator**
- **Code (P): Participant**
- **Number of participants present_______________**

| **Participant identifier number** | **Age** | **Years in role** | **Number of children** | **Educational status** | **Ethical consent, written** |
| --- | --- | --- | --- | --- | --- |
| **P1** |  |  |  |  |  |
| **P2** |  |  |  |  |  |
| **P3** |  |  |  |  |  |
| **P4** |  |  |  |  |  |
| **P5** |  |  |  |  |  |
| **P6** |  |  |  |  |  |
| **P7** |  |  |  |  |  |
| **P8** |  |  |  |  |  |
| **P9** |  |  |  |  |  |
| **P10** |  |  |  |  |  |
| **P11** |  |  |  |  |  |
| **P12** |  |  |  |  |  |
